# Unleashing creativity in people with Parkinson’s disease: a pilot study of a co-designed creative arts therapy

**DOI:** 10.1007/s00415-024-12878-0

**Published:** 2025-01-23

**Authors:** Blanca T. M. Spee, Nienke M. de Vries, Sara Zeggio, Marjoke Plijnaer, Jan-Jurjen Koksma, Annelien A. Duits, Thieme Stap, Gert Pasman, Suzanne Haeyen, Sirwan Darweesh, Julia Crone, Bastiaan R. Bloem, Matthew Pelowski

**Affiliations:** 1 Department of Neurology, Radboud University Medical Center, Donders Institute for Brain, Cognition and Behaviour, Nijmegen, the Netherlands; 2 Vienna Cognitive Science Hub, University of Vienna, Vienna, Austria; 3 Department of Cognition, Emotion, and Methods in Psychology, Faculty of Psychology, University of Vienna, Vienna, Austria; 4 Radboud University Medical Center Health Academy, Nijmegen, the Netherlands; 5 Department of Medical Psychology, Radboud University Medical Centre, Nijmegen, the Netherlands; 6 Department of Medical Psychology, Maastricht University Medical Centre, Maastricht, the Netherlands; 7 Art Unbound, collaboration partner of Radboud University Medical Center, Nijmegen, the Netherlands; 8 Fontys University of Applied Sciences, Research Group Professional Workplaces, Eindhoven, The Netherlands; 9 Faculty of Industrial Design Engineering, Delft University of Technology, Delft, the Netherlands; 10 GGNet, Centre of Expertise for Personality Disorders Apeldoorn, Centre for Mental Health, PO Box 2003, Scelta, 7230 GC Warnsveld the Netherlands; 11 Research Group Arts & Psychomotor Therapies in Health Care, Academy of Health & Vitality, HAN University of Applied Sciences, PO Box 6960, 6503 GL Nijmegen, the Netherlands

**Keywords:** Parkinson’s disease, Creative arts therapy, Art-based methods, Anxiety, Well-being

## Abstract

**Background:**

Conventional medical management, while essential, cannot address all multifaceted consequences of Parkinson’s disease (PD). This pilot study explores the potential of a co-designed creative arts therapy on health-related quality of life, well-being, and pertinent non-motor symptoms.

**Methods:**

We conducted an exploratory pilot study with a pre-post design using validated questionnaires. Eight individuals with PD participated in the program. The investigated intervention was a 10-week creative arts therapy with weekly 90–120-min sessions, guided by three creative therapists. Participants were allowed to autonomously select from multiple creative media based on their personal preferences. Explored co-primary outcomes included health-related quality of life (PDQ-39), well-being (ICECAP-A), anxiety/depression (HADS), executive functioning (BRIEF-A), resilience/mental flexibility (FIT-60), and self-efficacy (GSES). We used paired sample *t* tests for pre–post analysis of the co-primary outcomes and Wilcoxon signed-rank tests for PDQ-39 sub-scores. We also included aesthetic responsiveness (AReA) and healthcare consumption (IMCQ adapted for PD) questionnaires reported as descriptive statistics.

**Results:**

The results showed a significant reduction in anxiety and an increase in well-being. We also observed a slight improvement in cognitive functioning. Finally, we noted a reduction in healthcare consumption (fewer visits at neurologists, specialized PD nurses, and allied healthcare professionals).

**Conclusion:**

These findings cautiously suggest that our co-designed, multi-media creative arts therapy has the potential to increase well-being and reduce anxiety, while reducing healthcare consumption. These preliminary findings support the need for a larger, randomized controlled trial to explore the therapeutic potential of creative arts therapy in PD care.

**Supplementary Information:**

The online version contains supplementary material available at 10.1007/s00415-024-12878-0.

## Introduction

Parkinson’s disease (PD) is the worlds’ fastest growing neurodegenerative condition, affecting millions worldwide [1]. PD is characterized by motor dysfunctions such as tremor, bradykinesia, and stiffness [2]. Beyond these motor symptoms, PD also involves a wide range of non-motor features that profoundly affect the health-related quality of life and well-being of individuals [3, 4]. Anxiety and depression are particularly prevalent non-motor symptoms [5]. These motor and non-motor symptoms have debilitating consequences [6, 7], including diminished social engagement, autonomy, and self-efficacy—i.e., the belief in one’s ability to succeed in one’s own actions [8].

Conventional medical management, while essential, cannot address all multifaceted consequences of PD, especially non-motor symptoms that require a person-centered and lifestyle-oriented approach [9]. A multidisciplinary and holistic approach is likely needed, fueling interest to generate evidence for non-pharmacological interventions [10, 11]. Among these, interventions within creative arts therapy represent a promising approach [12–16]. For example, creative arts therapy in the visual art domain has been suggested to increase visual-cognitive skills and emotional well-being in people with PD, with improvements in emotional expression, social interaction, and overall life satisfaction [17, 18]. Music intervention have shown to have beneficial effects on motor function, speech, and mood, but also stigma and anxiety reduction [19]. Dance for people with PD increased executive functioning, spatial awareness, cognitive flexibility as well as reduced anxiety, depression, and apathy [20–22]. Positive effects of engaging in creative activities (including, e.g., dance, music, theater, visual arts) have also been described outside the PD field. Studies in older adults have reported, for example, increased well-being and personal growth, while community programs noted greater social engagement, less isolation, decreased experience of stigma, and better community ties [23–25]. These findings raise the intriguing question whether it is possible to investigate art just as if it were a drug, including a focus on efficacy, cost-effectiveness, and adverse effects [12].

To tackle this question, multiple challenges must be addressed. One relates to the apparent contrast between traditional biomedical approaches, which aim for generalizability and causality, and the more person-centered and lifestyle-oriented approaches of creative arts therapies or similar programs, which aim for creating meaningful care at an individual level [23, 26–31]. Existing studies in PD have been successful in showing beneficial effects on multiple health-related factors; however, they often focus on one specific creative domain, such as visual art, music, or dance [see for review, 12]. We believe for establishing a meaningful person-centered approach, it is crucial to fully embrace a broad spectrum of creative media tailored to the unique needs, abilities, and preferences of individuals with PD [32]. Research on person-centered arts-based interventions offering multiple creative media often lack scientific rigor, particularly due to the absence of adequately powered randomized control trials [12, 16, 33].

To address these multiple challenges, we have initiated a collaborative effort, drawing parallels to the structured phases of medical trials and incorporating the person-centered integrative approach of creative arts therapy [23, 32]. We have co-designed a specific creative arts therapy as an intervention together with people with PD, creative arts therapists, and researchers from various disciplines [36]. Our specific creative art therapy was a 10-week intervention allowing participants to autonomously select from multiple creative media, based on their personal preferences. Sessions were conducted weekly in an art atelier providing an artistically stimulating learning environment and multiple media [34, 35]. We termed this learning environment the ‘creative playground’ [36]. Continuous person-centered guidance from creative therapists ensured personalized care throughout the intervention period.

Through this participatory approach, we aimed to bridge medical and arts-based practices, ensuring that our intervention is both practical and meaningful for individuals with PD. By providing person with PD with a sense of ownership in designing and establishing PD care, we also hope to increase the chances of this therapy being effective in larger-scale trials to evaluate its relevance within PD care. Details of this co-design process are reported in a separate paper [36].

Here, we present the exploratory pilot phase and report the quantitative outcomes of our co-designed open label intervention study [36]. We assessed the effects on self-reported health-related quality of life, well-being, and other pertinent non-motor symptoms, providing a proof of concept that our intervention might lead to clinical improvements in PD. We also examined the impacts on healthcare consumption assuming that active engagement in creative activities might reduce healthcare strain. The findings will guide the design of future randomized trials and identify the primary health-related outcome measures.

## Methods

The study design was an explorative, mixed-method approach, using a self-reported questionnaire assessment before and after the intervention (pre–post design).

### Participants

Inclusion criteria were 18 years or older, diagnosed with PD, willingness to participate, and having signed informed consent. Eligible participants with a cognitive impairment (MoCA score < 18, [37]) were excluded as participants were required to be capable of completing the questionnaires.

### Procedure

Participants were invited through the outpatient clinic of the Radboudumc (Center of Expertise for Parkinson and Movement Disorders) and a large healthcare innovation project [38]. Interested individuals were first provided with an information letter detailing the study’s purpose and procedures. This was followed by a discussion with the researcher, allowing participants to ask questions and gain a thorough understanding of what participation entailed. Those who chose to participate then signed an informed consent form. Before starting the intervention, we conducted a baseline clinical assessment including standard demographics and PD-related health and disease state (MDS-UPDRS III [39], PD-related medication intake (see Table S3 in Supplementary Material), and global cognitive status using MoCA [37]). These assessments were performed in clinic by a certified researcher. We used paper–pencil for pre–post assessments, which could be completed at home (pre-assessment period: approximately 1 week before intervention start; post-assessment period: 4 weeks).

Based on our findings of a significant reduction in anxiety, we conducted a follow-up questionnaire to retrospectively collect data on psychotropic medication intake (see Table S3 in Supplementary Material).

This project was approved by ethical board METC Oost-Nederland, Radboudumc (file number; 2022–15919). As this was an explorative pilot study, we did not conduct a formal power analysis, and we aimed for a convenience sample of 8–12 participants.

### Intervention design—creative playground

The intervention design was co-designed with 14 people with PD, 4 creative arts therapists, and researchers trained in medicine (2 neurologist), 3 researchers trained in neuroaesthetics, 2 learning scientists, 1 neuropsychologist, and 1 expert in non-pharmacological interventions [36]. Five of the participants with PD only supported the design process at an earlier phase, including one patient researcher who advised us throughout the project. Nine participants helped to fine-tune the intervention design and filled out the questionnaires. Creative therapists were trained in multiple domains (i.e., a multi-media art therapist and artist with expertise in visual arts, fashion design, creative writing, drama, dance and acting; a music therapist with expertise in digital art (visuals, film, music); a dance/drama therapist). All were educated in PD including medication (side-) effects.

The co-designed intervention was a 10-week program with weekly 90–120-min sessions. Sessions were conducted in an art atelier with accessibility to multiple creative media, a space which we describe as a ‘creative playground.’ The weekly sessions started with an interactive performance with the participants or as a performance from the creative therapists (about 10–15 min), followed by participants’ self-determined creative activities, which could change during and between sessions. The sessions concluded with a shared reflection time, allowing participants to express their thoughts and experiences (5–10 min). Participants were guided by three creative therapists and encouraged to engage in multiple creative activities to explore, find, and express their own individual creativity.

### Stimuli—creative toolbox

Basic supplies of the creative sessions were:

- Visual arts: aquarelle, oil paints, chalk, pencils, fine and rough brushes, body painting supplies, clay or sculpturing supplies, photography equipment, polaroids, and filmmaking tools.
- Drama: a selection of clothes, masks, and props for role-playing.
- Movement and dance: open access to spaces and loudspeakers for bodily expressive activities; regular live music was provided at the beginning of the session.
- Creative writing: poetry encouraged with a variety of inspiring books, typewriters, and pen-inks.
- Music: instruments available included piano, guitar, various percussion tools, a microphone for singing, and a synthesizer.
- Additional crafts: sewing, cooking, clay work, toy figures, and wood construction sets with small building blocks.

Creative media were adapted between sessions if needed to address individual needs and wishes.

### Outcomes

The explored outcomes included *PD-specific health-related quality of life* (PDQ-39 [40, 41]) and *well-being* (ICECAP-A [42, 43]). We further included pertinent healthcare measures, specifically *anxiety* and *depression* (HADS [44]), *resilience/mental flexibility* (FIT-60 [45]), *self-efficacy* (GSES [44]), *aesthetic responsiveness* (AReA [46]), and *subjective executive functioning* (BRIEF-A [47]). The BRIEF-A features two main indices: the *metacognitive index*, which assesses an adult’s planning, organizing, and problem-solving capabilities using active working memory; and the *behavioral regulation index*, which evaluates an adult’s control over behaviors and emotional responses. Finally, we used a healthcare consumption questionnaire (IMCQ adapted for PD [48]) to explore potential shifts or reductions in healthcare consumption. This was assessed by absolute number of visits (in person or through tele-support) at neurologists, specialized PD nurses, and visits at allied healthcare professionals (i.e., general practitioner, social worker, physio-, occupational-, and speech therapist, dietitian, homeopath or acupuncturist, psychologist, occupational health physician, home care assistant).

### Statistical analysis

Statistical analysis was conducted using RStudio (Version 2023.12.0 + 369). We used descriptive statistics to present the distribution of participants’ demographic characteristics and other variables of interest in our dataset. For within-subject comparisons of PDQ-39 [40, 41], ICECAP-A [42, 43], HADS [44], FIT-60 [45], GSES [44], and BRIEF-A [47], we conducted paired sampled *t* tests. For the PDQ-39 sub-scores, we applied Wilcoxon signed-rank test as normal distribution was not assumed. Chosen alpha level was set to 0.05. Results of the AReA [46] and IMCQ adapted for PD (healthcare consumption [48]) are reported as descriptive statistics. Specifically, healthcare consumption of participants was assessed through the absolute number of visits, with pre-measures covering the last 2 months before the intervention and post-measures covering the full 10-week period of the intervention. Finally, we visualized probability density and summary statistics using raincloud plots (R package raincloud plots [49]) for pre- and post-intervention data, alongside mean and confidence intervals.

## Results

Our study involved nine participants. One participant was excluded from the analysis due to incomplete post-questionnaire responses. Data were analyzed from eight participants. We report demographics and baseline clinical characteristics in Table 1.

**Table 1:** Standard demographics and baseline clinical characteristics (*N* = 8)

Characteristics	Absolute numbers (% of total) or mean and standard deviation	Range
Age—years	54.50 ± 9.01	44–67
Age of diagnosis—years	47.75 ± 10.61	28–65
Gender identity—no. (%)	3 (37.50%) men	
Ethnicity: Dutch	8 (100%)	
Educational level—no. (%)	*n* = 1 (12.50%) university degree *n* = 5 (62.50%) higher professional education *n* = 2 (25.00%) secondary vocational education	
MD-UPDRS scores	**Mean and standard deviation**	**Range**
Part I	14.38 ± 7.58	5–24
Part II	13.25 ± 9.07	5–26
Part III	37.75 ± 20.95	15–62
HY-stage	*n* = 2, HY-stage 1 *n* = 3, HY-stage 2 *n* = 2, HY-stage 3 n = 1, HY-stage 4	1–4
Global cognition	**Mean and standard deviation**	**Range**
MoCA	28.12 ± 1.96 (range 5)	25–30
Levodopa Equivalent Daily Dosage—in mg	814.5 ± 441.6	188–1293

Full report of means and standard deviations of each (pre-post) measurement are reported in Table S1 in Supplementary Material. Violin plots not reported in the main manuscript are shown in Figs. S1-S6. Results of the health-related quality of life sub-scores are reported in Table S2. Results of the within-subject comparison are reported in Table 2.

**Table 2:** Within-subject comparison of healthcare related factors

	Pre			Post			95% CI				
	*M*	*SD*	Range	*M*	*SD*	Range	*t*(7)	Lower	Upper	*p* value	Cohen’s *d*
Quality of life											
PDQ39-SI	35.14	8.97	24.95- 52.55	32.93	11.42	21.04- 48.96	1.35	−1.66	6.07	0.219	2.21
Well-being											
Total score	0.84	0.07	0.73–0.92	0.90	0.03	0.85–0.94	– 2.97	– 0.12	0.01	0.021	1.16
Anxiety and depression											
Anxiety	7.13	2.64	3–11	5.25	2.12	2–8	3.64	0.66	3.09	0.008	0.78
Depression	4.63	2.77	1–9	4.25	2.49	1–8	0.89	– 0.62	1.37	0.402	0.14
Subjective executive functioning											
Behavioral Regulation	54.25	11.97	35–74	54.43	14.11	46–77	−0.12	−5.98	5.41	0.906	0.01
Meta-cognition	64.00	9.09	49–75	60.00	9.40	47–75	1.95	−0.84	7.41	0.099	0.43
Mental flexibility											
Total score	216.63	40.43	164–286	221.88	38.55	157–283	−0.64	−24.62	14.12	0.542	0.13
Self-efficacy											
Total score	29.88	4.82	23–37	31.00	4.17	25–38	– 1.14	−3.47	1.22	0.293	0.25

**Fig. 1:**
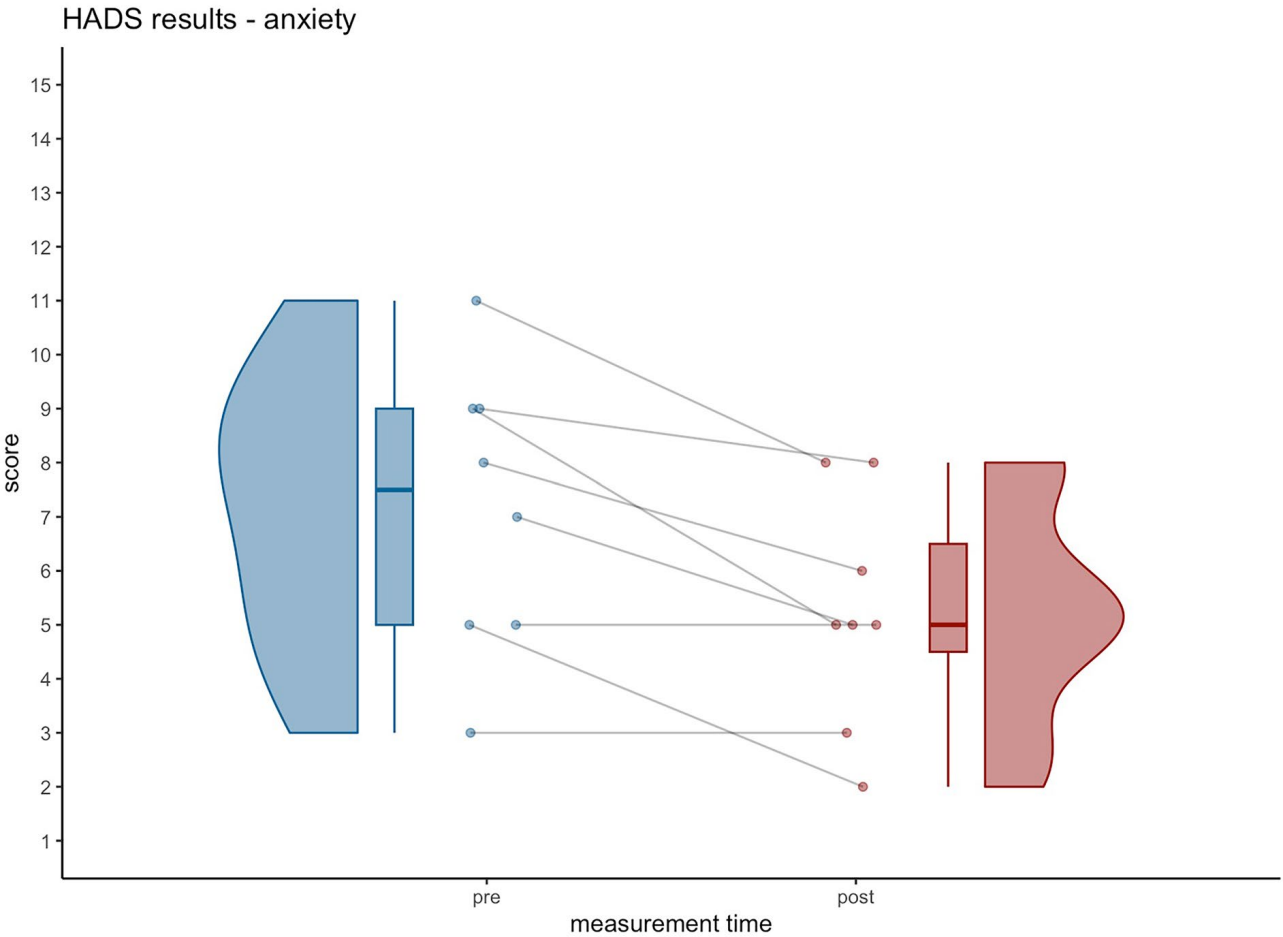
Significant decrease in anxiety from pre (7.13 ± 2.64) to post (5.25 ± 2.12). Mean difference 1.88. Note: lower scores indicate an improvement (threshold sub-score values: 0–7 no anxiety, 8–10 borderline abnormal, 11–21 abnormal anxiety). One participant improved from abnormal anxiety levels (clinical case) to borderline abnormal; two improved from borderline abnormal to normal levels; three reduced anxiety levels, including one borderline case; two cases showed no anxiety with no change pre–post

The most notable finding was a significant reduction in anxiety, as indicated by the decrease in the HADS anxiety sub-score (see Table 2). The mean reduction of 1.88 is clinically relevant, as it exceeds the suggested minimal clinically important difference (MCID) of 1.50 [7, 50]. Three participants exhibited a clinically relevant reduction (abnormal anxiety to borderline abnormal to normal anxiety), achieving lower HADS anxiety sub-score (see Fig. 1).

**Fig. 2:**
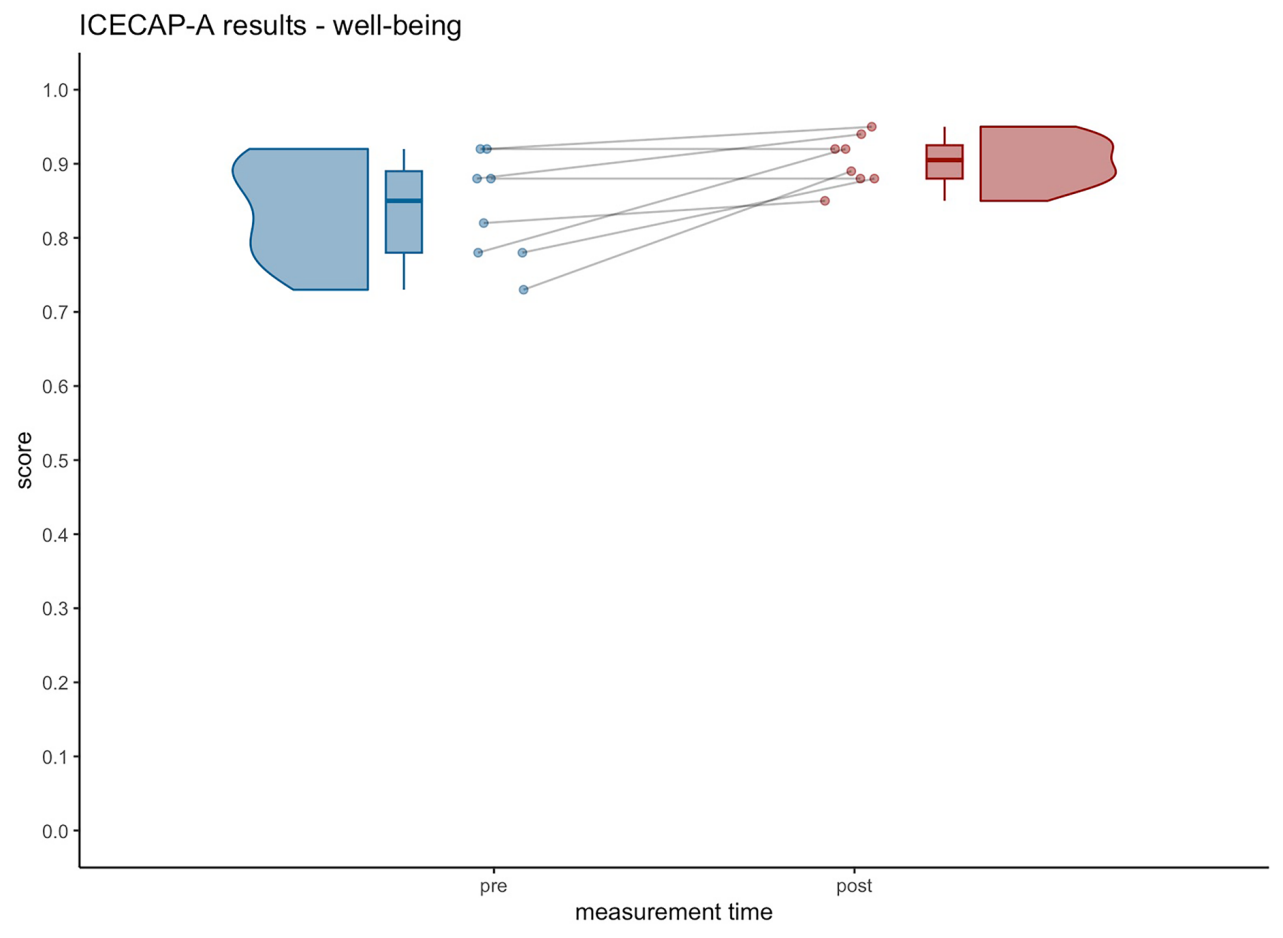
Significant increase in well-being from pre (0.84 ± 0.07) to post (0.90 ± 0.03). Note: higher scores indicate an improvement

*Well-being* showed a significant increase (see Table 2). Violin plots visualize increase on participant well-being scores as well as probability density (see Fig. 2).

Considering the other explored outcomes, we found a slight increase in the *meta-cognition index* (MD = 10.00, sub-score of *executive functioning*, see Table 2) and ‘stigma’ (MD = 4.43, sub-score of health-related quality, see Table S1). We did not find significant or notable changes in other sub-scores of *health-related quality of life, depression*, *executive functioning* sub-score *behavioral regulation*, *resilience and mental flexibility, self-efficacy*, and *aesthetic responsiveness*.

Finally, the intervention period saw a total decrease of 30 healthcare visits, with appointments specifically involving neurologists and specialized PD nurses reducing from 16 to 10 visits. Other healthcare visits included physiotherapists (*n* = 15 visits) and homeopaths (*n* = 11 visits), as well as one visit each to an occupational therapist and a speech therapist.

## Discussion

Our pilot study investigated the integration of creative arts therapy within PD care, yielding notable observations. We recorded a significant reduction in anxiety and an increase in well-being. In addition, there were slight improvements in meta-cognition and fewer healthcare visits to neurologists, specialized PD nurses, and mainly to physiotherapists and homeopaths.

Anxiety is a prevalent and debilitating issue of PD [3, 5, 51] and is recognized as one of the primary stress-related neuropsychiatric symptoms [5, 52]. Our findings showed a significant decrease in anxiety, which is consistent with prior findings in creative arts therapy research [12]. In addition, our results might also be supported by studies in the field of both mindfulness and cognitive behavioral therapies, both of which have shown to be effective in reducing anxiety [7, 52–54]. Mindfulness, defined as moment-to-moment non-judgmental awareness [55], may also complement creative activities that not only engage a state of mindfulness—or even a flow experience [56]—but also activate creative cognition [57]. The creative multi-media nature of our intervention, emphasizing flexibility and tailored engagement, likely facilitated benefits similar to those seen in mindfulness or behavioral therapy, though creative arts therapy, particularly our patient-developed multi-media approach [36], is less studied [12]. In addition, creative activities might be particularly beneficial for people with PD, given the role of dopamine and brain regions involved in creative cognition, which are affected in PD [58–60]. Our approach may have also contributed to the slight improvements in meta-cognition and reduced healthcare utilization. Therefore, we strongly advocate for further research, and suggest that future larger, controlled studies should consider anxiety as a primary outcome.

Considering the long-term sustainability of the observed effects, it is plausible that the benefits, particularly reductions in anxiety and increase in well-being, could persist if participants continue engaging in creative activities post-intervention. Longitudinal studies or long-term adherence measurement timepoints are needed to confirm the durability of these effects over extended periods.

Regarding the source of the beneficial effects on anxiety, it is essential to consider both the impact of the creative arts therapy and the social cohesion it fosters. While the creative activities themselves likely play a significant role in reducing anxiety by providing an outlet for expression and engagement, the social interactions and sense of community developed during the sessions may also contribute substantially. Even though future studies might consider disentangling these factors by comparing arts-based interventions with and without structured social components, proving this might be challenging. Even more so, the social component might be intrinsic and a key feature of creative arts therapies. Hence, future studies might also consider announcing the social and communal aspects of creative arts therapies as fundamental to their therapeutic impact and investigate the balance between and importance of both, the social and the artistic elements.

Creative arts therapies in PD care have primarily been explored through interventions focusing on single media, particularly music, dance, and visual arts [see for review, 12]. Structured visual art interventions demonstrated improvements in quality of life and visual-spatial skills after two weekly sessions over 10 weeks [17, 18]. Similarly, music and dance therapies report beneficial effects: group guitar classes studied in a randomized control trial held twice weekly for 6 weeks led to significant improvements in anxiety, mood, and quality of life, with lasting benefits in mood and anxiety [61], while a dance intervention with weekly sessions over 22 weeks showed improvements in self-esteem, quality of life, and motor symptoms [22]. In contrast, our co-designed, multi-media ‘creative playground’ intervention allowed participants flexible access to multiple media—including visual arts, music, drama, and movement on music—within a shorter, 10-week timeframe with only weekly sessions [36]. Despite fewer sessions, our study observed clinically relevant reductions in anxiety and increases in well-being. Our pilot study cautiously suggests that a multi-media approach fostering individualized creative expression may offer comparable benefits more effectively. Future research could further explore the impact of media variety, session frequency, and intervention duration to determine optimal formats for creative arts therapies in PD care.

As for the target population, creative arts therapy should ideally be offered to all individuals with PD, given the broad spectrum of benefits observed. However, certain subgroups might derive stronger advantages, such as those experiencing significant non-motor symptoms like anxiety and depression, stress, and individuals with early to moderate stages of PD who can actively engage in the sessions. People who lack intrinsic motivation for creative activities or having issues in mastering mindfulness skills might also be suitable candidates for such programs, offering them a therapeutic field which they might not have considered themselves.

A consideration for future studies is the potential interaction between dopaminergic medication, specifically dopamine agonists, and creative arts therapy [62, 63]. While dopaminergic medications primarily target motor symptoms, they can also influence mood and cognitive functions, potentially affecting the benefits of creative arts interventions [4, 64]. Case reports and empirical studies suggest that dopaminergic treatment may increase the tendency to engage in creative activities [62, 63]. Conversely, creative activity itself might complement the effects of dopaminergic treatments [4, 65, 66]. We suggest that careful guidance is needed to support people with PD to find the right balance in their creative activities—i.e., individual creativity dosage—along with their standard treatment, as adverse effects such as impulsive and compulsive behavior may arise [4, 64]. Even though four participants had high levodopa equivalent daily dosages (> 1.000mg per day, see for full report Table S3 in Supplementary Material), the creative therapists did not note any adverse behavior during the sessions. Nonetheless, future research should explore these interactions to optimize holistic person-centered therapeutic approaches offering multiple healthcare disciplines, including creative therapy [9, 12, 38].

Our finding of reduced anxiety raises the question of whether psychotropic medications influenced these outcomes. To explore this, we conducted a follow-up questionnaire asking participants retrospectively about their intake of medications that affect mood, or drugs specifically for anxiety and depression. Data were obtained from seven of the eight participants (see Table S3 in the Supplementary Material). The participant who did not provide information had already exhibited normal anxiety levels at baseline and showed no change post-intervention. Among the seven respondents, only one had been taking psychotropic drugs prior to and during the intervention and showed a transition from borderline to normal anxiety levels after the intervention. The others, who also showed a reduction in anxiety, were not taking any psychotropic medications but only drugs for PD. Based on these observations, we feel confident that the significant reduction in anxiety can largely be attributed to the intervention itself, rather than to psychotropic medication use. Nonetheless, future studies should assess psychotropic and other medication use to further corroborate the intervention’s effects.

Finally, we want to note that the small sample size, absence of a control group, and the exploratory nature of this study necessitate cautious interpretation of our findings and highlight the need for further detailed exploration. Despite these limitations, the promising findings suggest that our co-designed intervention, as a multi-media creative arts therapy, merits more extensive examination through randomized controlled trials.

## Supplementary Information

Below is the link to the electronic supplementary material.

Supplementary file1 (DOCX 816 KB)

## Data Availability

The dataset used in this study is available from the corresponding author upon request.
