# Supplementary material for: Unleashing creativity in people with Parkinson’s disease: a pilot study of a co-designed creative arts therapy
Source: J Neurol. 2025 Jan 23;272(2):161. doi: 10.1007/s00415-024-12878-0 (PMC11758163; doi:10.1007/s00415-024-12878-0)

# Supplementary Material

**Unleashing Creativity in People with Parkinson’s disease:
A Pilot Study of a Co-Designed Creative Arts Therapy**

Blanca T.M. Spee^1,2,3,*^, Nienke M. de Vries^1^, Sara Zeggio^1^, Marjoke Plijnaer^7^, Jan-Jurjen Koksma^4^, Annelien A. Duits^5,6^, Thieme Stap^4,8^, Gert Pasman^9^, Suzanne Haeyen^10,11^, Sirwan Darweesh^1^, Julia Crone^2^, Bastiaan R. Bloem^1^, Matthew Pelowski^2,3^

^1^ Radboud university medical center, Donders Institute for Brain, Cognition and Behavior; Department of Neurology, Centre of Expertise for Parkinson & Movement Disorders, Nijmegen, the Netherlands

^2^ Vienna Cognitive Science Hub, University of Vienna, Vienna, Austria

^3^ Department of Cognition, Emotion, and Methods in Psychology, Faculty of Psychology, University of Vienna, Vienna, Austria

^4^ Radboud university medical center Health Academy, Nijmegen, the Netherlands

^5^ Department of Medical Psychology, Radboud University Medical Centre

^6^ Department of Medical Psychology, Maastricht University Medical Centre

^7^ Art Unbound, collaboration partner of Radboud university medical center, Nijmegen, the Netherlands

^8^ Fontys University of Applied Sciences, Research Group Professional Workplaces, Eindhoven, The Netherlands

^9^ Faculty of Industrial Design Engineering, Delft University of Technology, Delft, the Netherlands
^10^ GGNet, Centre for Mental Health, Scelta, Centre of Expertise for Personality Disorders Apeldoorn, PO Box 2003, 7230 GC Warnsveld, the Netherlands

^11^ Research Group Arts & Psychomotor Therapies in Health Care, Academy of Health & Vitality, HAN University of Applied Sciences, PO Box 6960, 6503 GL Nijmegen, the Netherlands

* Corresponding Author: [blanca.spee@radboudumc.nl](mailto:blanca.spee@radboudumc.nl)

Keywords: Parkinson’s disease, creative arts therapy, art-based methods, anxiety, well-being

Tables

**Table S1.** Descriptive Statistics of all factors reported along mean and standard deviation (SD); pre-post improvements are adapted to scale directionality, to show improvements as positive and decline as negative mean difference (MD).

| Factors | mean | standard deviation | pre-post comparison mean |
| --- | --- | --- | --- |
| **quality of life (decrease is improvement, pre-post)** | | |  |
| pre_PDQ39_mobility | 42.81 | 25.62 |  |
| pre_PDQ39_dailylife_activities | 40.10 | 23.35 |  |
| pre_PDQ39_emotional_wellbeing | 32.81 | 11.45 |  |
| pre_PDQ39_stigma | 27.87 | 18.46 |  |
| pre_PDQ39_social_support | 22.92 | 22.16 |  |
| pre_PDQ39_cognitive_impairment | 34.38 | 18.60 |  |
| pre_PDQ39_communication | 32.29 | 16.33 |  |
| pre_PDQ39_bodily_discomfort | 47.92 | 17.11 |  |
| post_PDQ39_mobility | 40.00 | 24.71 | 2.81 |
| post_PDQ39_dailylife_activities | 38.02 | 23.82 | 2.08 |
| post_PDQ39_emotional_wellbeing | 30.73 | 16.05 | 2.08 |
| post_PDQ39_stigma | 23.44 | 16.95 | 4.43 |
| post_PDQ39_social_support | 23.96 | 13.68 | -1.04 |
| post_PDQ39_cognitive_impairment | 31.25 | 13.36 | 3.13 |
| post_PDQ39_communication | 28.13 | 21.79 | 4.17 |
| post_PDQ39_bodily_discomfort | 47.92 | 10.68 | 0.00 |
| pre_PDQ39_PDQ_SI | 35.14 | 8.97 | 2.21 |
| post_PDQ39_PDQ_SI | 32.93 | 11.42 | 2.81 |
| **well-being (increase is improvement, post-pre)** | | |  |
| pre_ICECAP_tot_scores | 0.84 | 0.07 | 0.65 |
| post_ICECAP_tot_scores | 0.90 | 0.03 | 0.69 |
| **anxiety and depression (decrease is improvement, pre-post)** | | |  |
| pre_HADS_subscore_anxiety | 7.13 | 2.64 |  |
| post_HADS_subscore_anxiety | 5.25 | 2.12 | 1.88 |
| pre_HADS_subscore_depression | 4.63 | 2.77 |  |
| post_HADS_subscore_depression | 4.25 | 2.49 | 0.38 |
| **subjective executive functioning (decrease is improvement, pre-post)** | | |  |
| pre_BRIEF_A_inhibition | 58.50 | 14.06 |  |
| pre_BRIEF_A_flexibility_in_problem_solving | 56.25 | 12.52 |  |
| pre_BRIE_A_emotion_regulation | 52.38 | 11.22 |  |
| pre_BRIEF_A_self_evaluation | 47.25 | 11.27 |  |
| pre_BRIEF_A_taking_initative | 61.13 | 9.66 |  |
| pre_BRIEF_A_working_memory | 64.50 | 9.72 |  |
| pre_BRIEF_A_planning_and_organisation | 61.63 | 8.99 |  |
| pre_BRIEF_A_task_evaluation | 62.50 | 6.16 |  |
| pre_BRIEF_A_orderliness | 59.50 | 14.00 |  |
| post_BRIEF_A_inhibition | 54.43 | 14.43 | 4.07 |
| post_BRIEF_A_flexibility_in_problem_solving | 58.57 | 11.01 | -2.32 |
| post_BRIEF_A_emotion_regulation | 53.14 | 12.97 | -0.77 |
| post_BRIEF_A_self_evaluation | 49.43 | 11.62 | -2.18 |
| post_BRIEF_A_taking_initative | 56.43 | 10.81 | 4.70 |
| post_BRIEF_A_working_memory | 60.86 | 8.15 | 3.64 |
| post_BRIEF_A_planning_and_organisation | 59.00 | 10.68 | 2.63 |
| post_BRIEF_A_task_evaluation | 58.00 | 7.21 | 4.50 |
| post_BRIEF_A_orderliness | 57.86 | 12.33 | 1.64 |
| pre_BRIEF_A_behavioral_regulation_index | 54.25 | 11.97 |  |
| pre_BRIEF_A_metacognition_index | 64.00 | 9.09 |  |
| post_BRIEF_A_behavioral_regulation_index | 54.43 | 14.11 | -0.18 |
| post_BRIEF_A_metacognition_index | 60.00 | 9.40 | 4.00 |
| **resilience/mental flexibility (comparison with population, post-pre)** | | |  |
| pre_FIT60_1_acceptence | 34.75 | 8.58 |  |
| pre_FIT60_2_diffusion | 30.50 | 12.52 |  |
| pre_FIT60_3_self_as_context | 31.38 | 8.78 |  |
| pre_FIT60_4_presence | 33.13 | 11.17 |  |
| pre_FIT60_5_value | 46.38 | 7.63 |  |
| pre_FIT60_6_acting | 40.50 | 7.03 |  |
| post_FIT60_1_acceptence | 35.13 | 6.83 | 0.38 |
| post_FIT60_2_diffusion | 31.75 | 13.01 | 1.25 |
| post_FIT60_3_self_as_context | 31.88 | 4.29 | 0.50 |
| post_FIT60_4_presence | 37.38 | 9.20 | 4.25 |
| post_FIT60_5_value | 46.38 | 7.65 | 0.00 |
| post_FIT60_6_acting | 39.38 | 6.89 | -1.13 |
| pre_FIT60_0_psychological_flexibility | 216.63 | 40.43 | 181.50 |
| post_FIT60_0_psychological_flexibility | 221.88 | 38.55 | 190.13 |
| **self-efficacy (increase is improvement, post-pre)** | | |  |
| pre_gses_totalscore | 29.88 | 4.82 |  |
| post_gses_totalscore | 31.00 | 4.17 | 1.13 |
| **aesthetic responsiveness (increase is improvement, post-pre)** | | |  |
| pre_area_1_aesthetic_appreciation | 18.00 | 6.99 |  |
| pre_area_2_intense_aesthetic_experience | 4.13 | 3.94 |  |
| pre_area_3_creative_behavior | 5.25 | 4.23 |  |
| post_area_1_aesthetic_appreciation | 19.00 | 5.61 | 1.00 |
| post_area_2_intense_aesthetic_experience | 4.63 | 3.74 | 0.50 |
| post_area_3_creative_behavior | 6.38 | 3.58 | 1.13 |

**Table S2.** Within subject comparison of healthcare related factors, specifically quality of life measured along the PDQ-39 sub-scores.

|  | pre | | post | |  |  |  | |
| --- | --- | --- | --- | --- | --- | --- | --- | --- |
|  | *M* | *SD* | *M* | *SD* | *V* | *p*-value | | *r* |
| **quality of life** |  |  |  |  |  |  | |  |
| mobility | 42.81 | 25.62 | 40.00 | 24.71 | 25.00 | .360 | | 0.96 |
| daily life activities | 40.10 | 23.35 | 38.02 | 23.82 | 11.50 | .343 | | 0.97 |
| emotional wellbeing | 32.81 | 11.45 | 30.73 | 16.05 | 18.50 | .882 | | 0.88 |
| stigma | 27.87 | 18.46 | 23.44 | 16.95 | 13.00 | .672 | | 0.56 |
| social support | 22.92 | 22.16 | 23.96 | 13.68 | 9.00 | .832 | | 0.65 |
| cognitive impairment | 34.38 | 18.60 | 31.25 | 13.36 | 18.00 | .548 | | 0.81 |
| communication | 32.29 | 16.33 | 28.13 | 21.79 | 13.00 | .672 | | 0.62 |
| bodily discomfort | 47.92 | 17.11 | 47.92 | 10.68 | 11.00 | .668 | | 0.84 |

Note: Sub-scores comparison was analyzed using a Wilcoxon signed-rank test and *r* as effect size.

**Table S3.** Levodopa Equivalent Daily Dose (LEDD) for each participant.

| **Participants** | **Drug-type** | **Drug intake  (amount in mg x frequency per day)** | **Total LEDD (in mg)** |
| --- | --- | --- | --- |
| 1 | PD-related drugs | Sinemet (62.5 x 3) | 188 |
|  | psychotropic medication^*^ | none |  |
| 2 | PD-related drugs | Sinemet (125 x 6)  Sinemet CR (125 x 1) | 844 |
|  | psychotropic medication^*^ | none |  |
| 3 | PD-related drugs | Madopar (125 x 9) | 1,125 |
|  | psychotropic medication^*^ | none |  |
| 4 | PD-related drugs | Sinemet (187.5 x 6) | 1,125 |
|  | psychotropic medication^*^ | NA |  |
| 5 | PD-related drugs | Madopar disper (125 x 6)  Ropinirol (10 x 2) | 1,150 |
|  | psychotropic medication^*^/treatment | none / deep brain stimulation |  |
| 6 | PD-related drugs | Symmetrel (100 x 2)  Ropinirol CR (8 x 1)  Duodopa (50 x 1)  Sinemet CR (250 x1) | 603 |
|  | psychotropic medication^*^ | none |  |
| 7 | PD-related drugs | Sinemet (62.5 x 3) | 188 |
|  | psychotropic medication^*^ | none |  |
| 8 | PD-related drugs | Sinemet (250 x 4)  Sinemet CR (250 x 1)  Sifrol | 1,293 |
|  | psychotropic medication^*^ | Citalopram (8 x 1)  Amitriptylin (10 x 2) |  |

Note. Two participants show low to moderate LEDD values (<400mg); two participants moderate LEDD values (<1000mg), and four participants high LEDD values (>1000mg). *Data of the psychotropic medication intake were collected retrospectively based on our findings of a significant reduction in anxiety.

**Figures**

**Figure S1.** Raincloud plots. Health-related quality of life; PDQ-39, total score. Note: reduction is improvement.


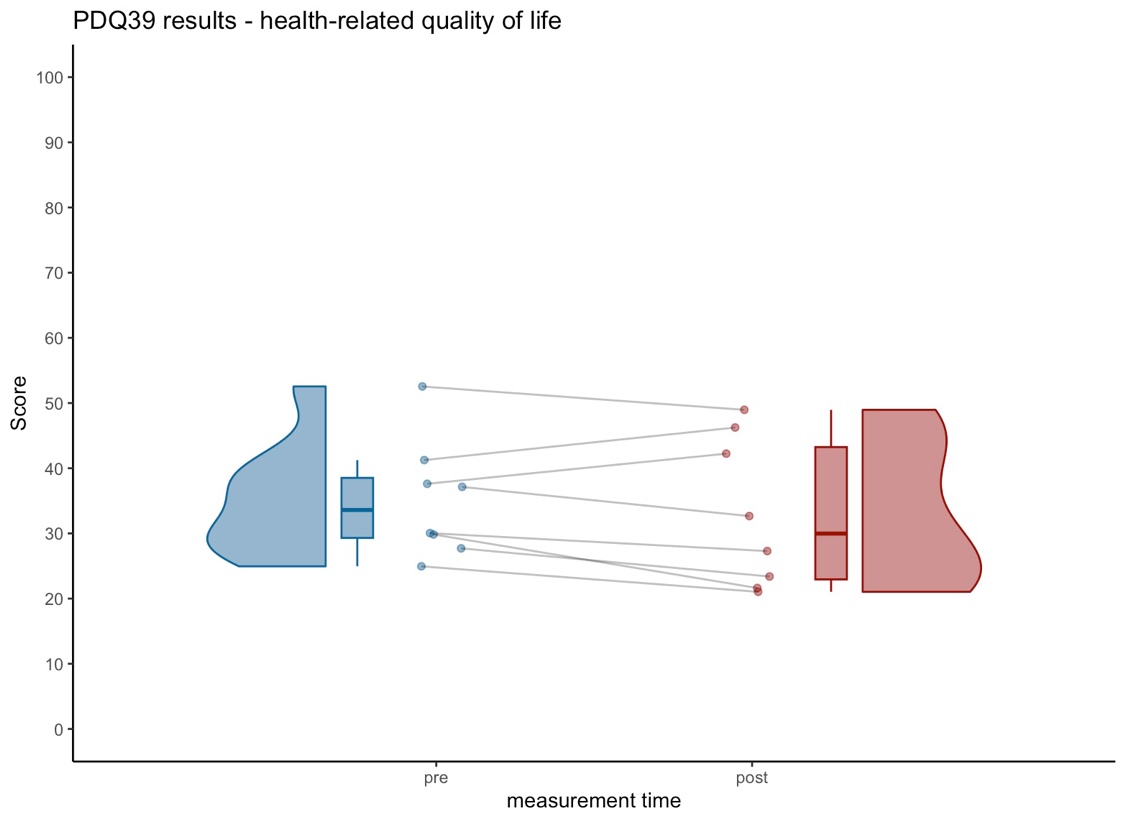


**Figure S2.** Raincloud plots. Depression; HADS, depression score. Note: reduction is improvement.

**
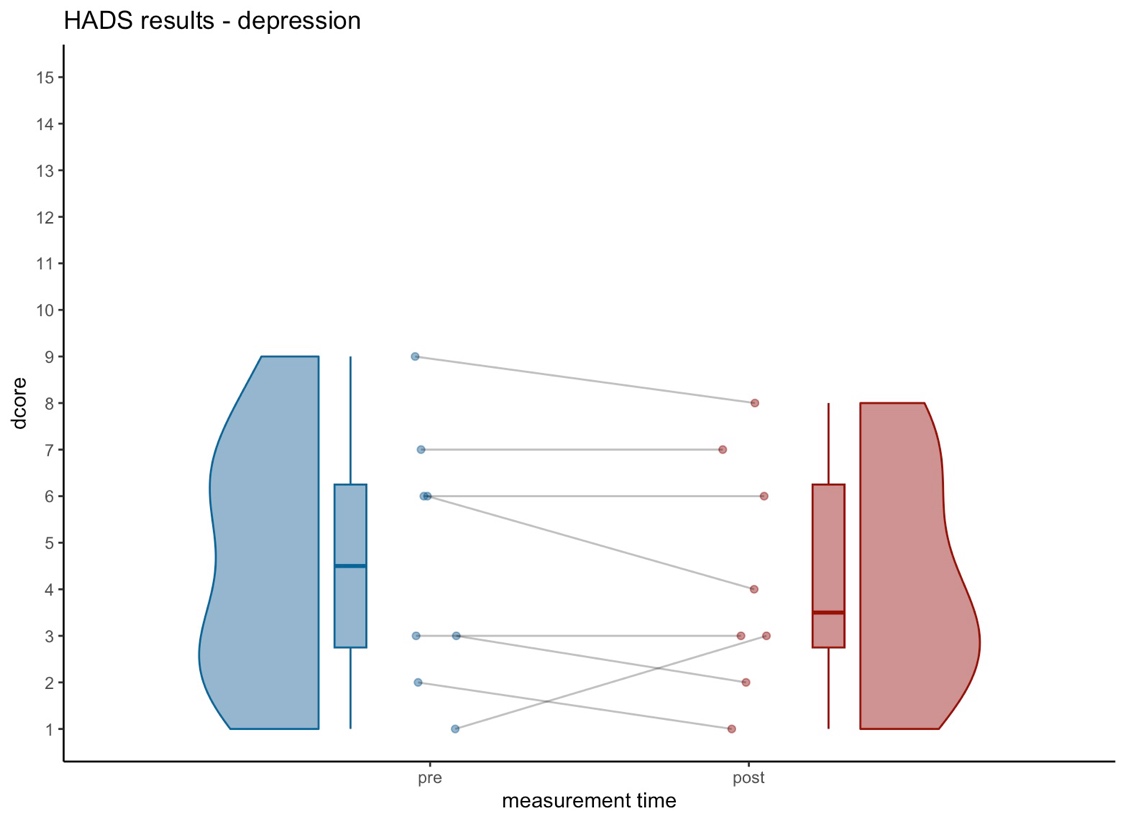
Figure S3.** Raincloud plots. Subjective executive functioning; BRIEF-A, (a) behavioral regulation and (b) metacognition total scores. Note: reduction is improvement.

(a)


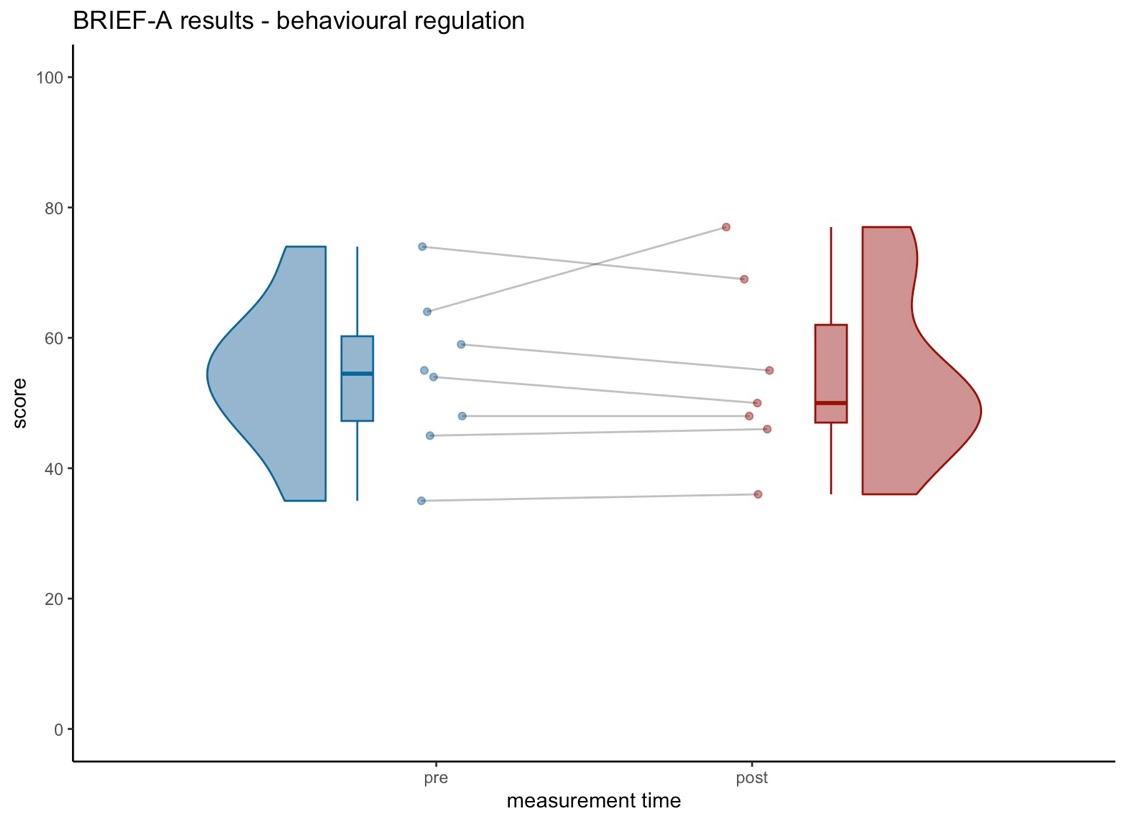


(b)


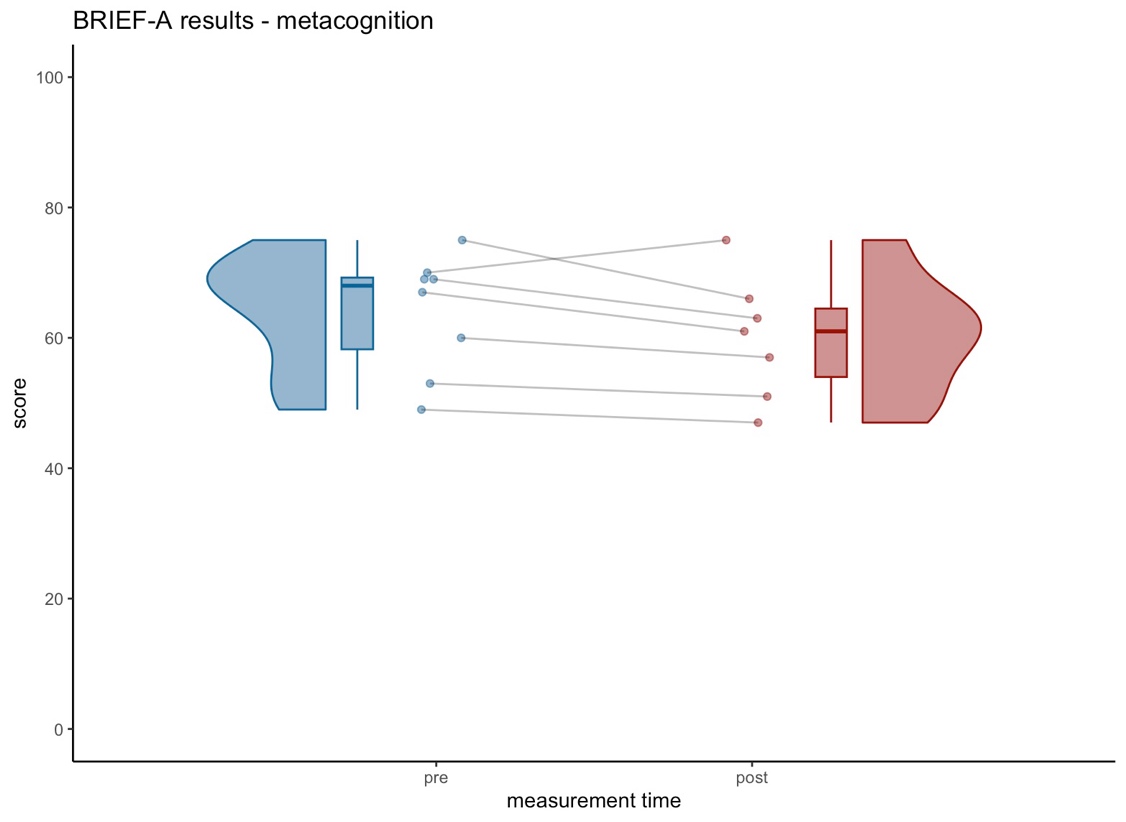


**Figure S4.** Raincloud plots. Mental flexibility; FIT60, total score. Note. increase is improvement considering group of comparison (Dutch population without neurological conditions, see also <https://www.actinactie.nl/flexibiliteits-index-test-fit-60/>).


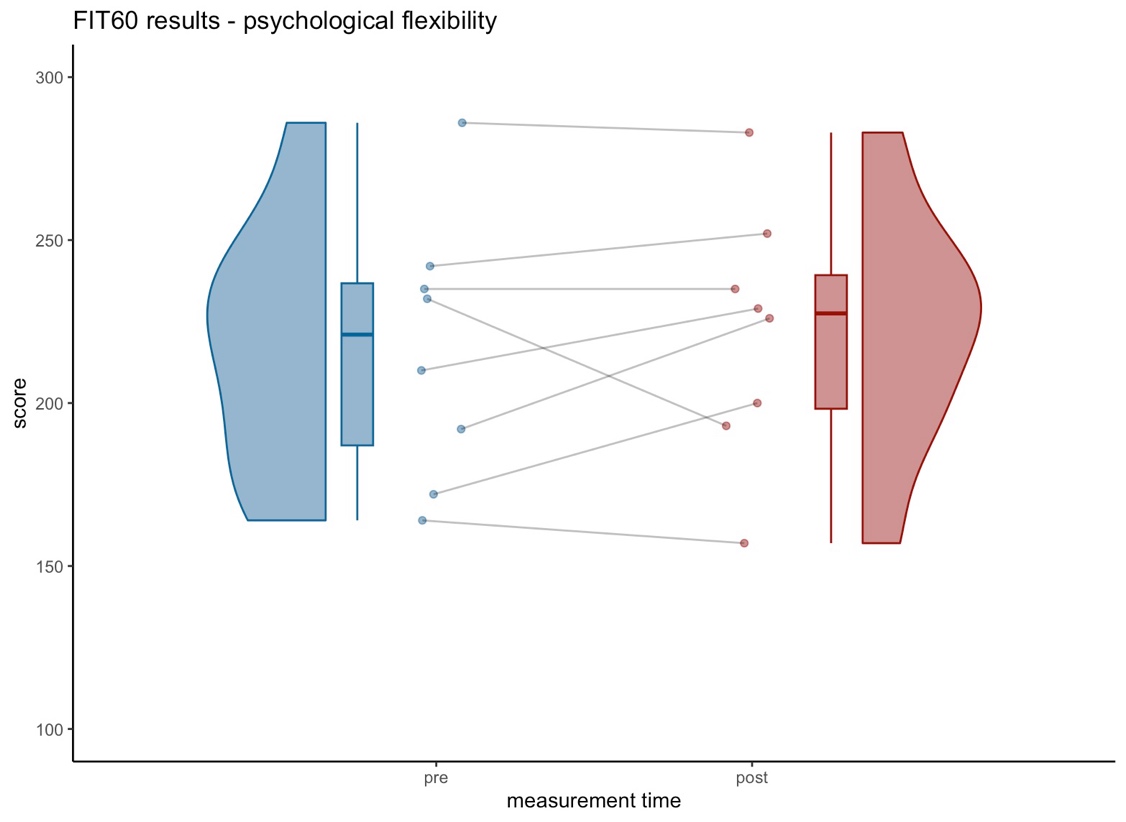


**Figure S5.** Raincloud plots. Self-efficacy; GSES, total score. Note: increase is improvement.


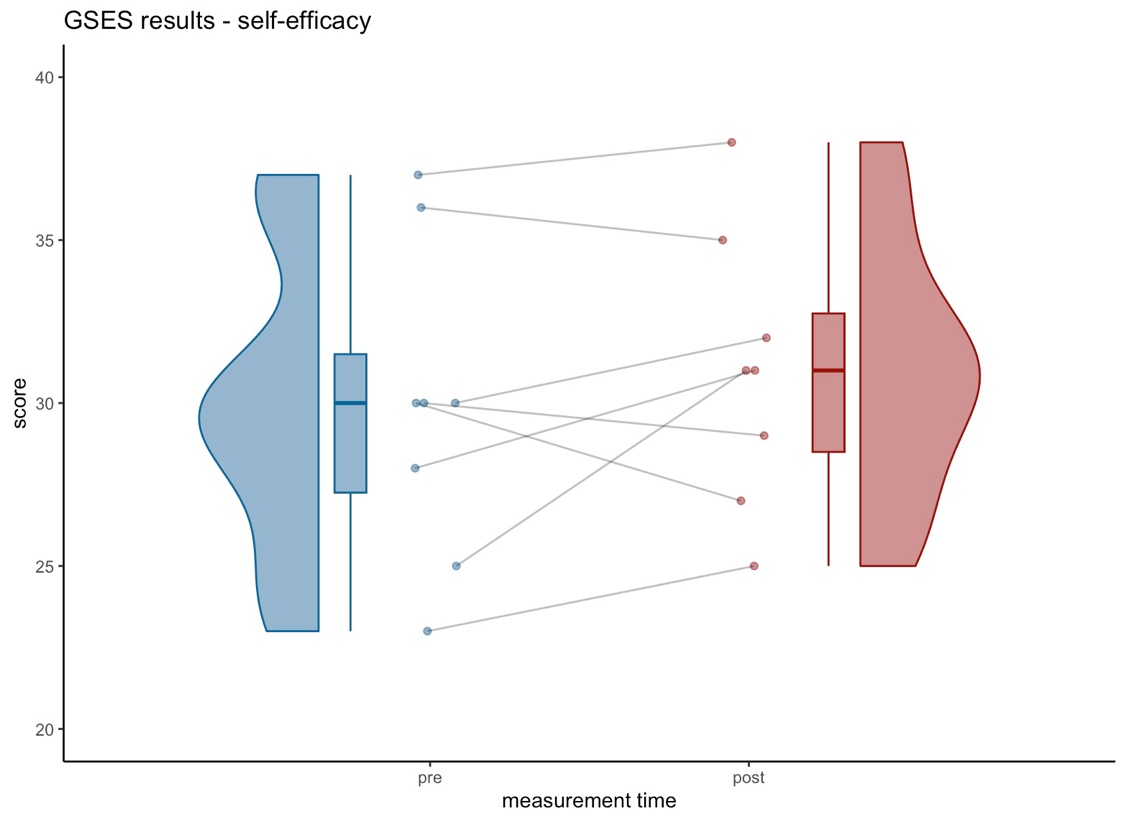


**Figure S6.** Raincloud plots. Aesthetic Responsiveness Assessment; AReA, sub-scores (a) aesthetic appreciation, (b) aesthetic experience, and (c) creativity. Note: increase is improvement.

(a)


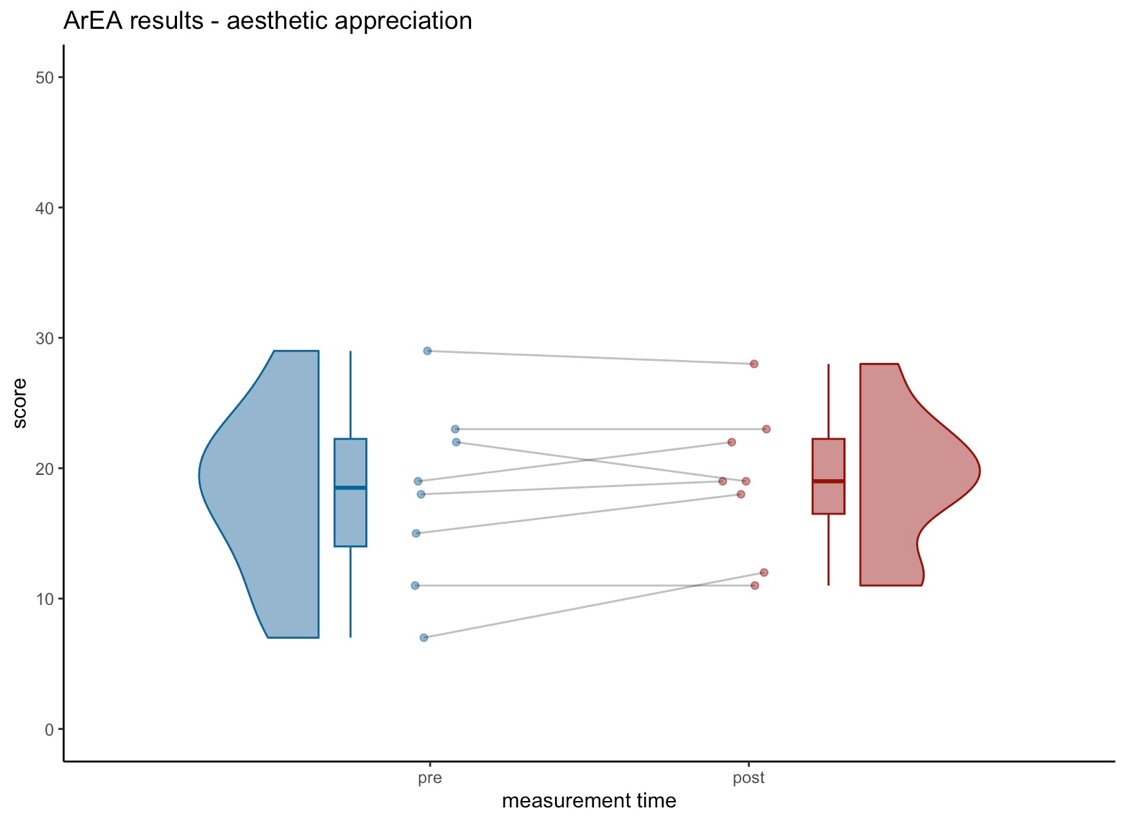


(b)


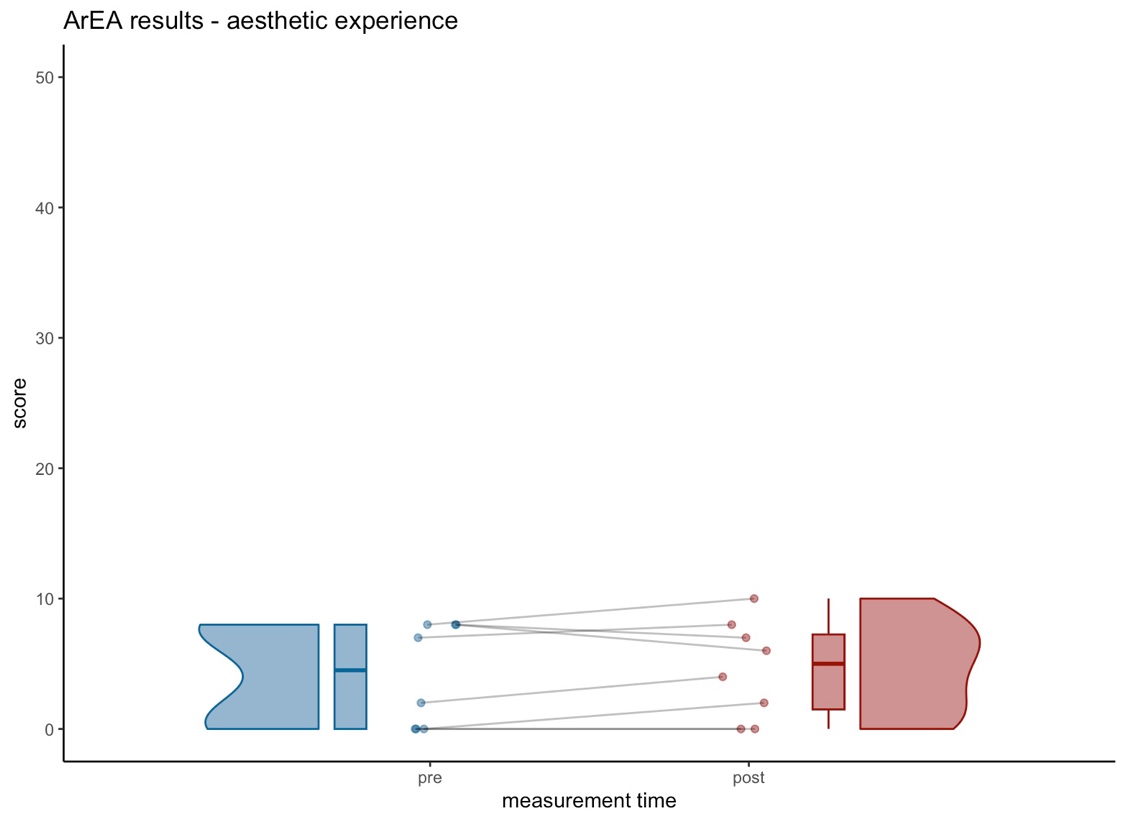


(c)


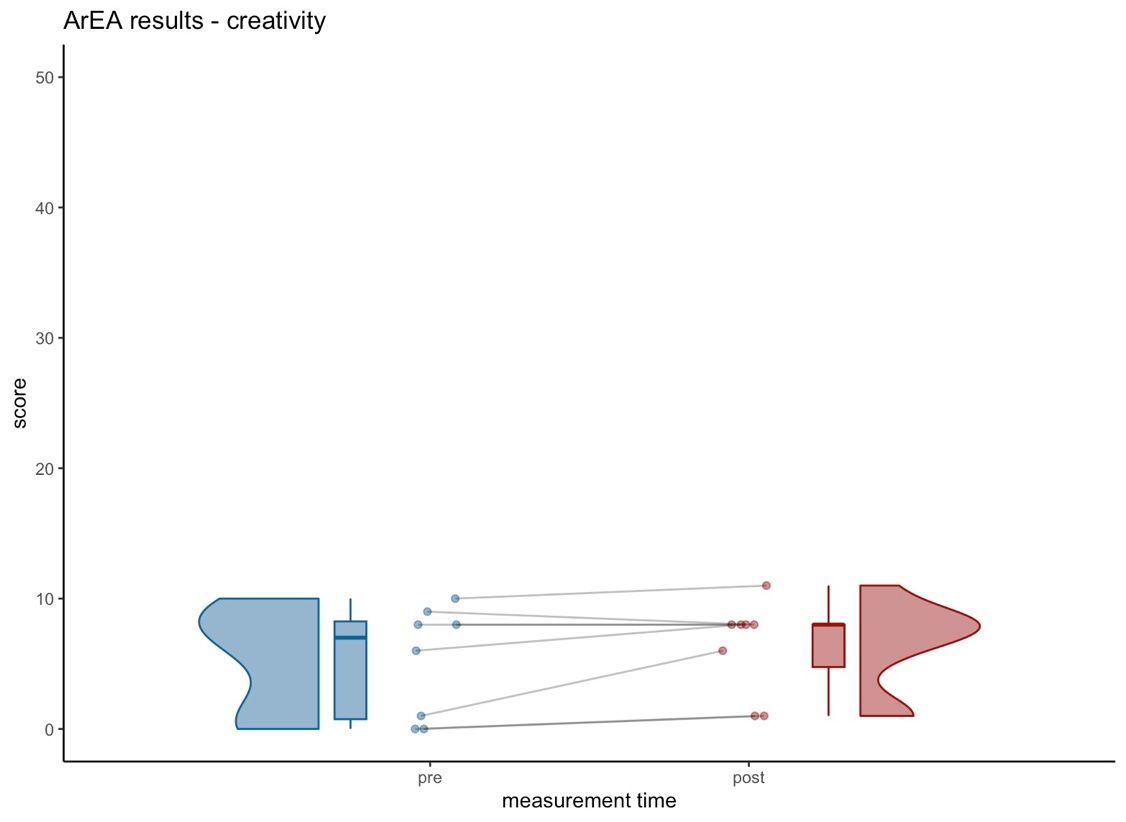

Supplement: Supplementary file 1 — Supplementary file1 (DOCX 816 KB) [file 415_2024_12878_MOESM1_ESM.docx]
